# Supplementary figures and images for: Four-Dimensional Characterization of the Babesia divergens Asexual Life Cycle, from the Trophozoite to the Multiparasite Stage
Source: mSphere. 2020 Oct 14;5(5):e00928-20. doi: 10.1128/mSphere.00928-20 (PMC7565898; doi:10.1128/mSphere.00928-20)

**a**

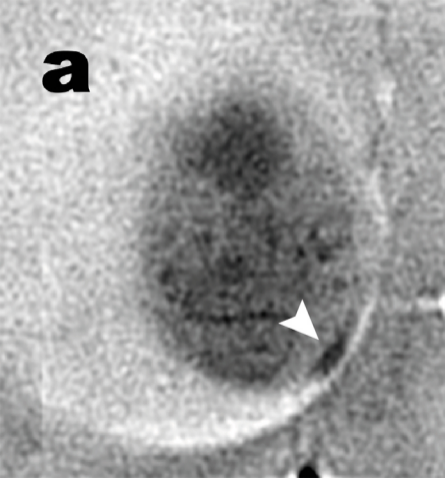

**b**

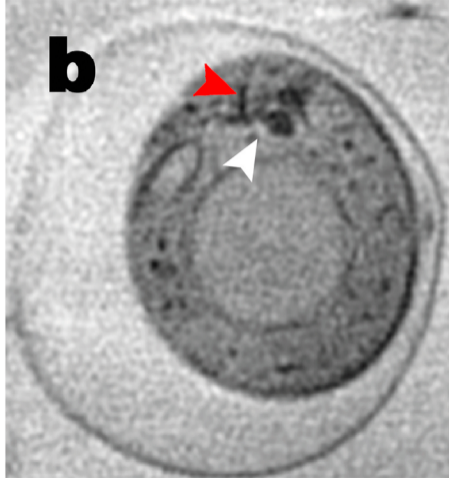

**c**

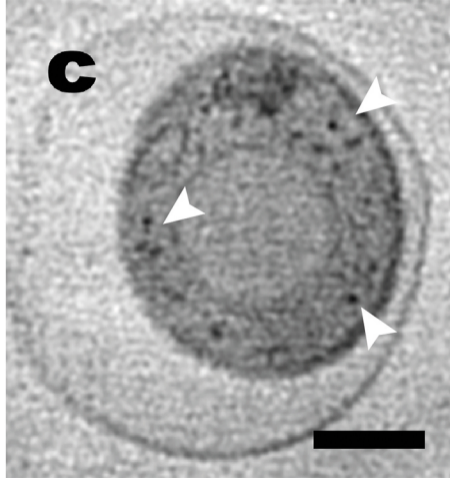

Supplement: FIG S1 [file mSphere.00928-20-sf001.pdf]

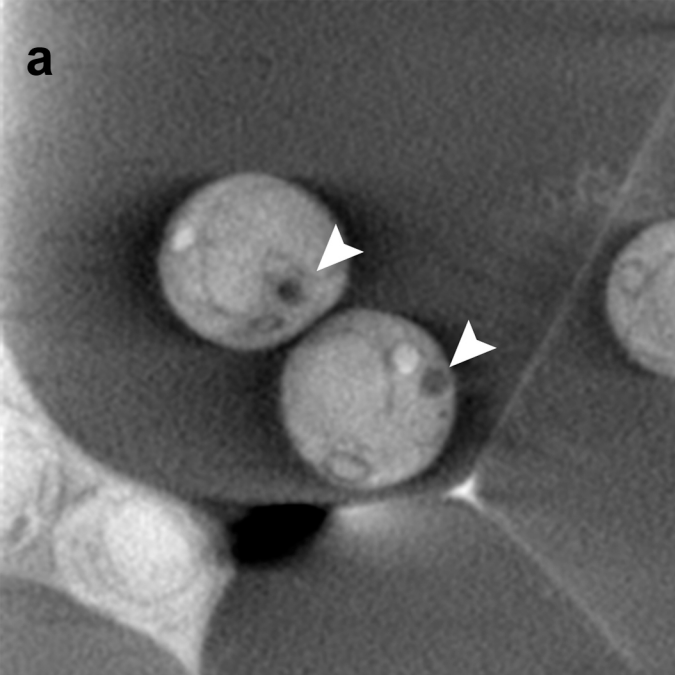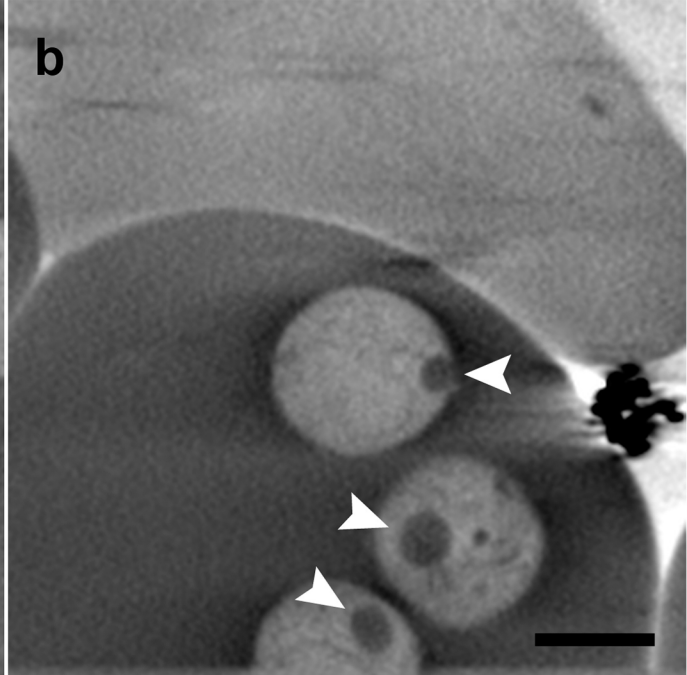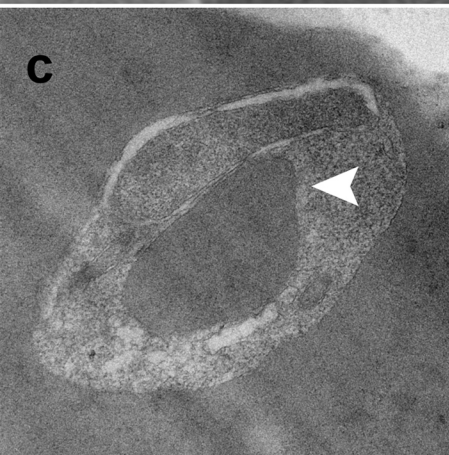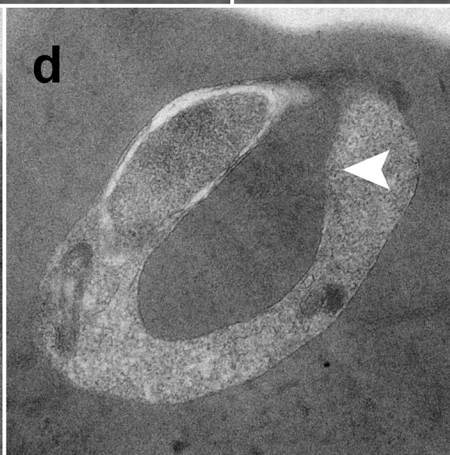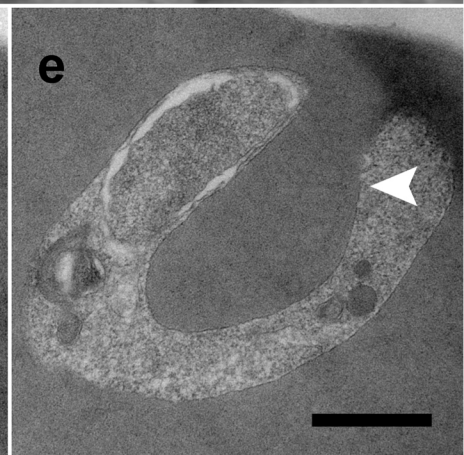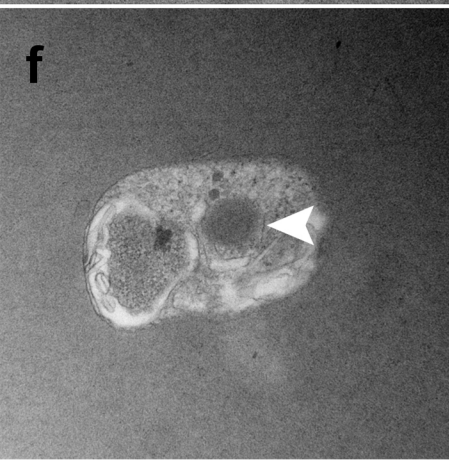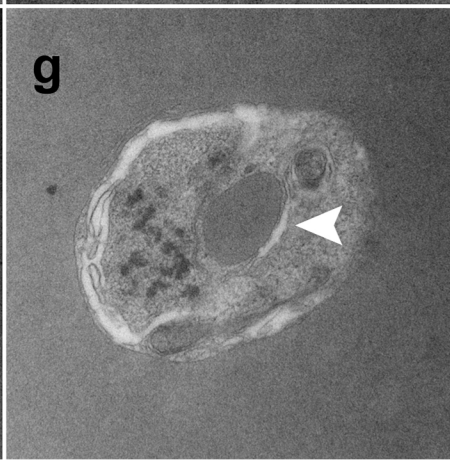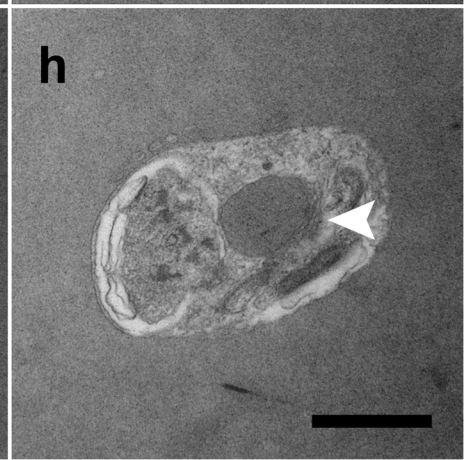

Supplement: FIG S2 [file mSphere.00928-20-sf002.pdf]

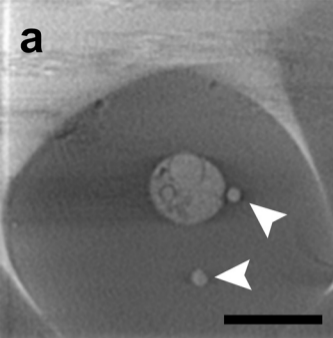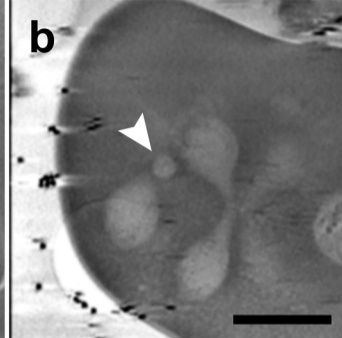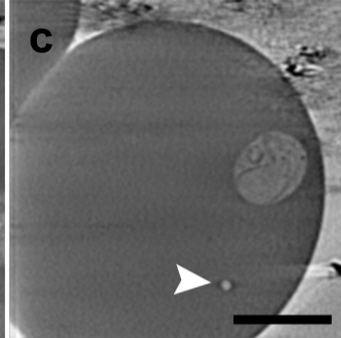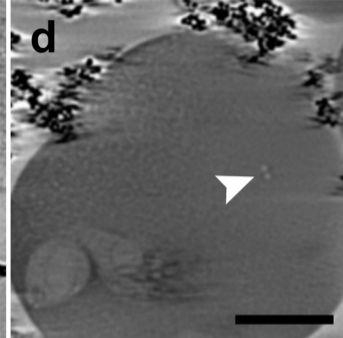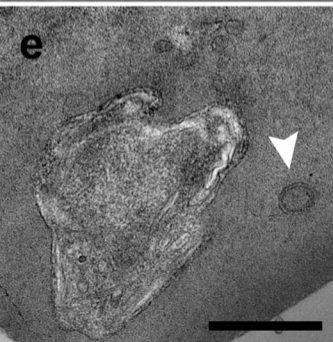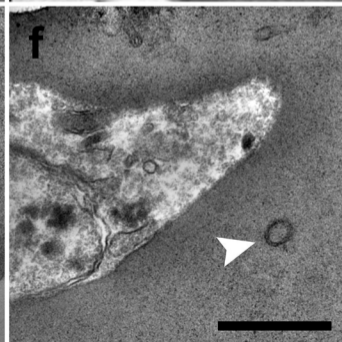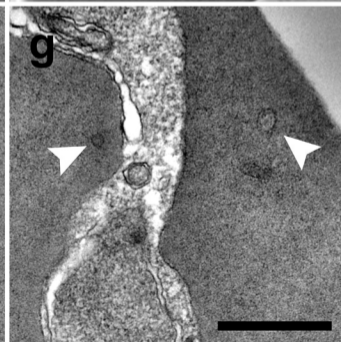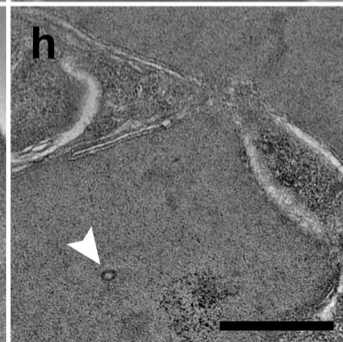

Supplement: FIG S3 [file mSphere.00928-20-sf003.pdf]

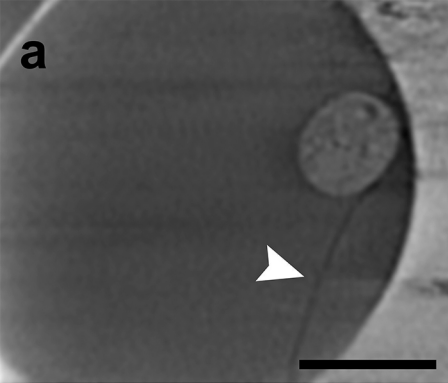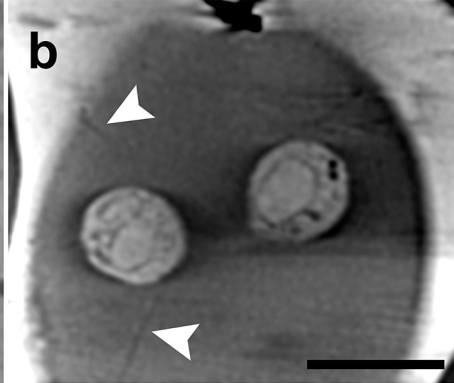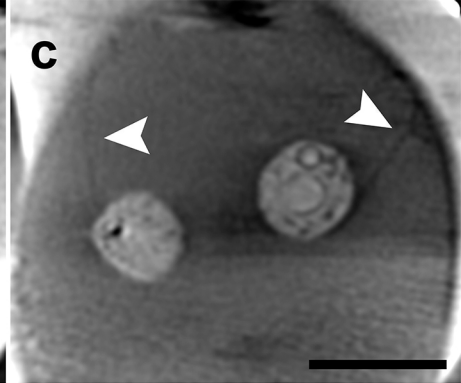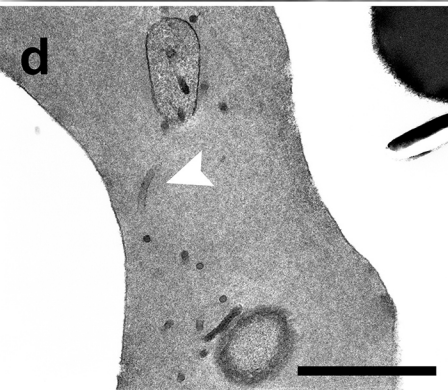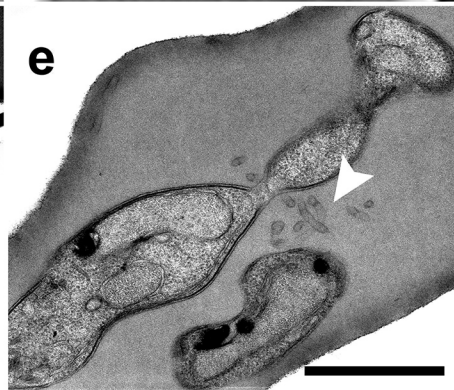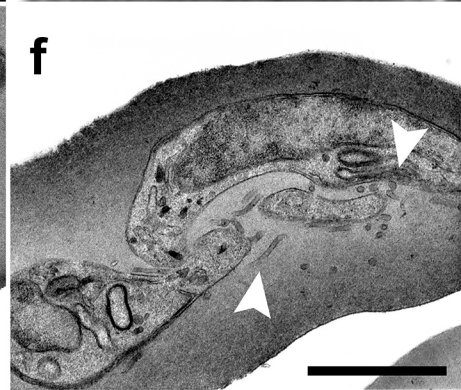

Supplement: FIG S4 [file mSphere.00928-20-sf004.pdf]

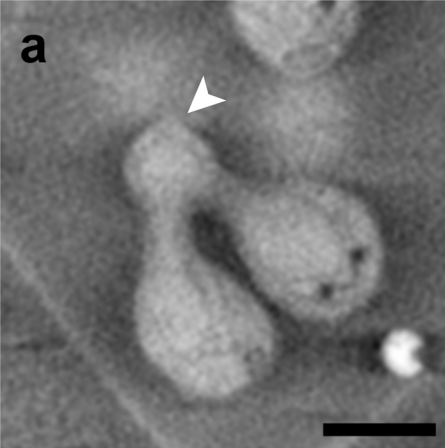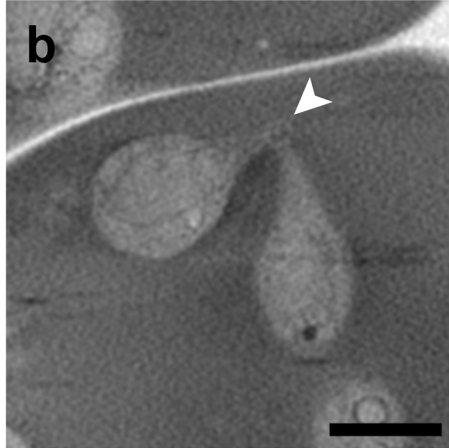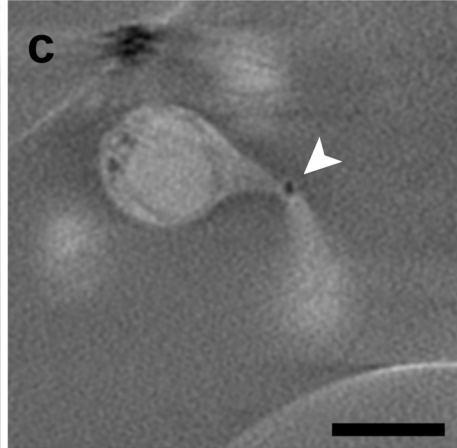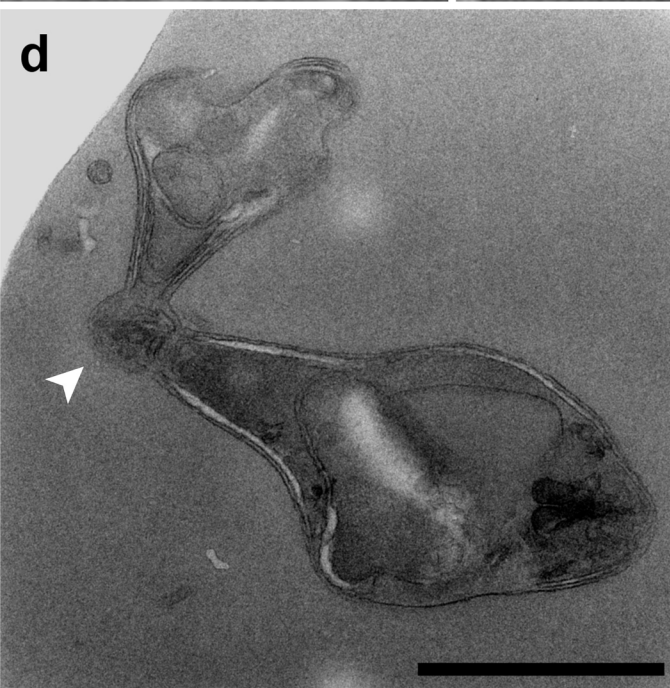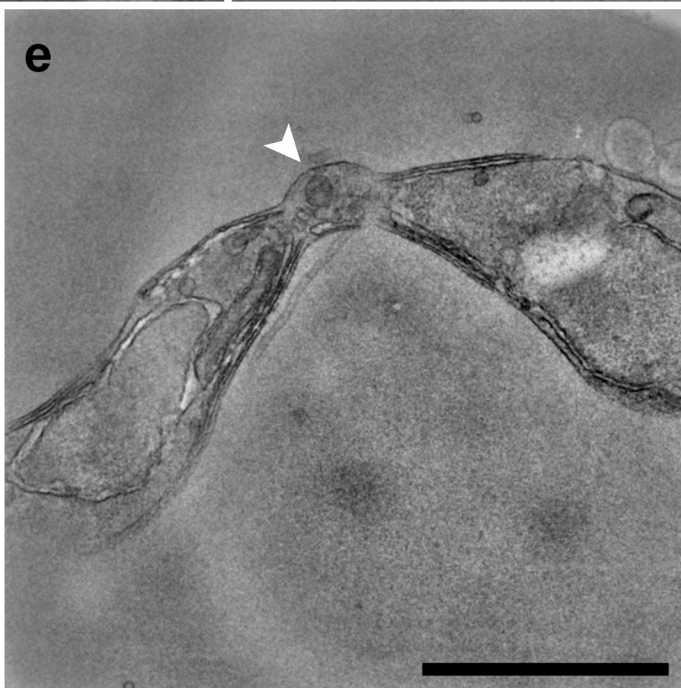

Supplement: FIG S5 [file mSphere.00928-20-sf005.pdf]

**a**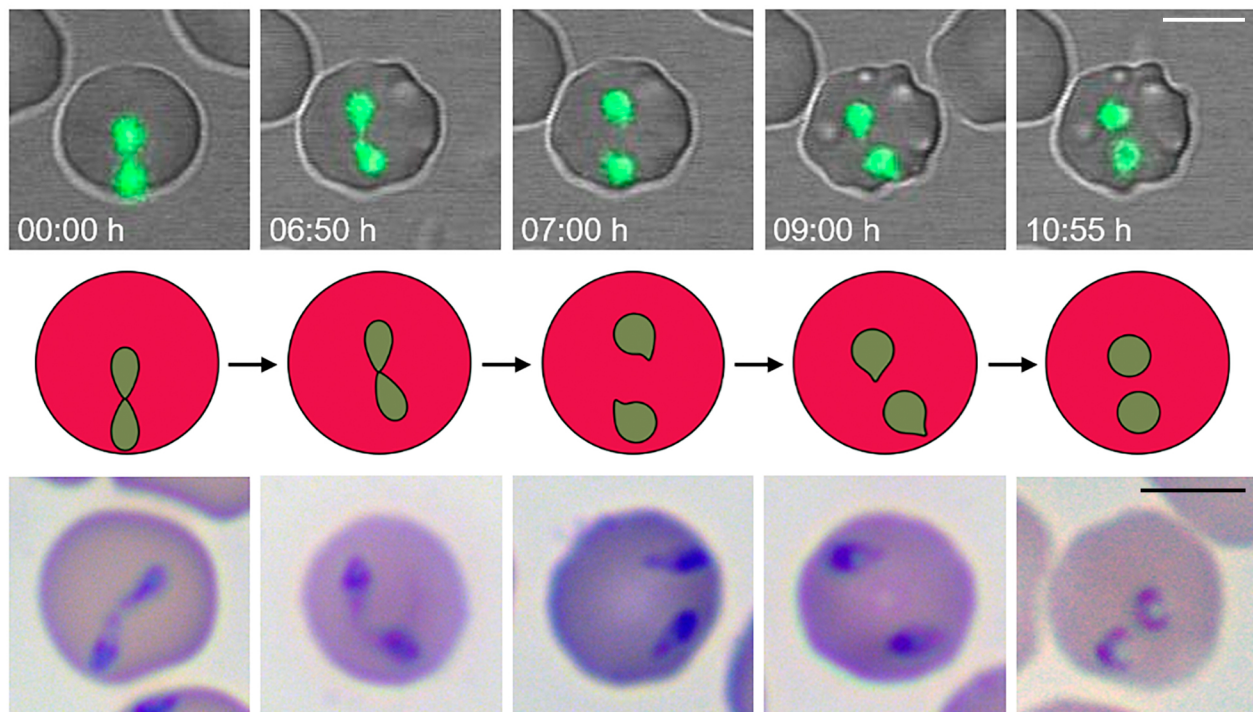**b**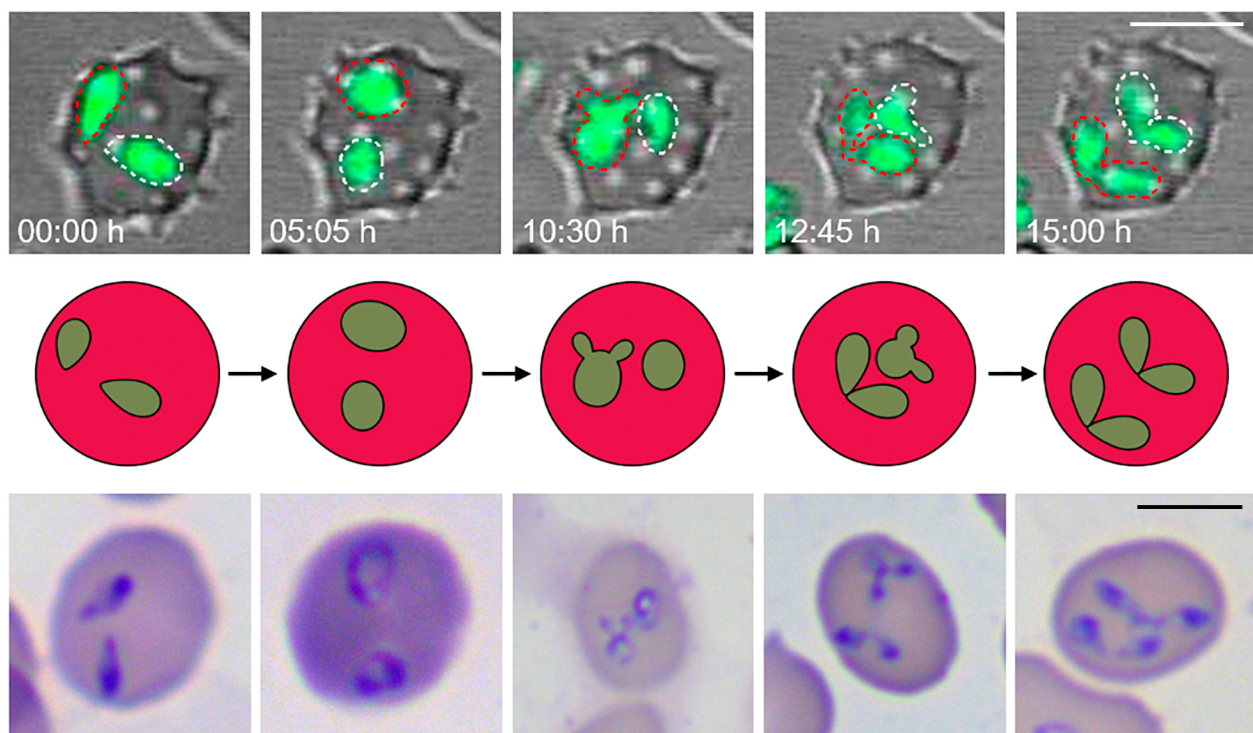

Supplement: FIG S6 [file mSphere.00928-20-sf006.pdf]

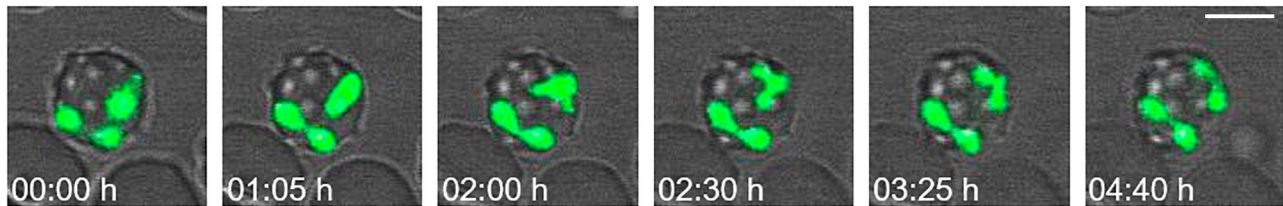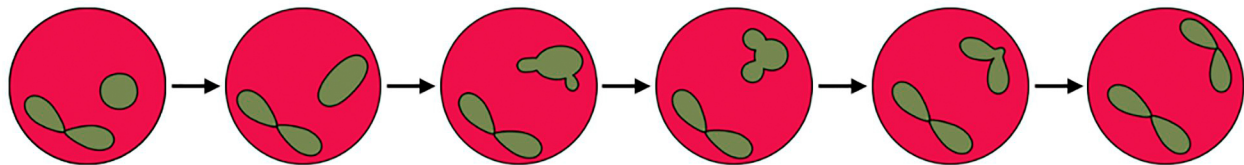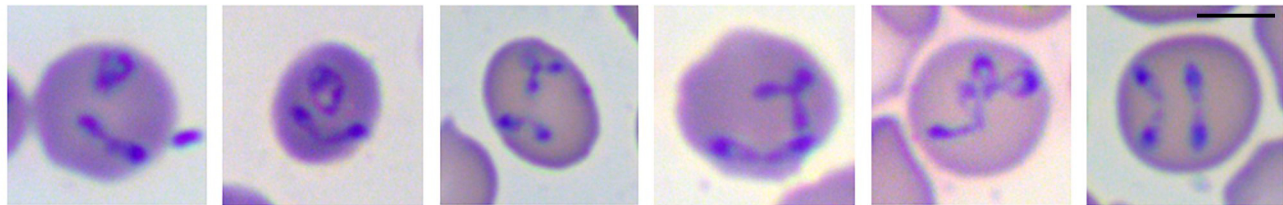

Supplement: FIG S7 [file mSphere.00928-20-sf007.pdf]

**a**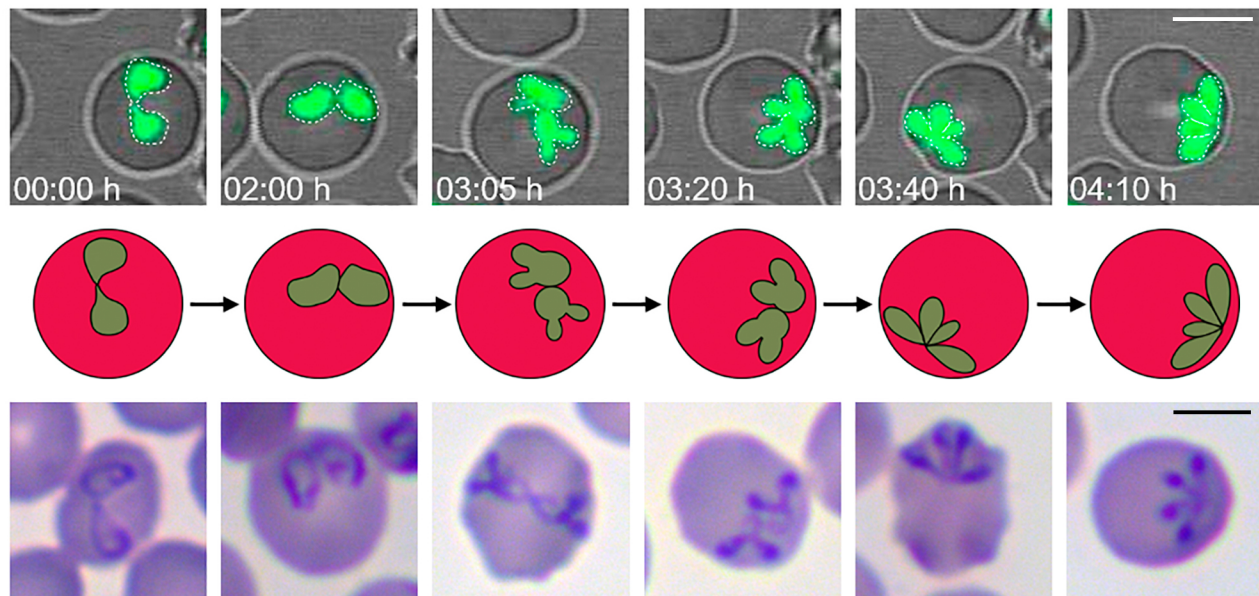**b**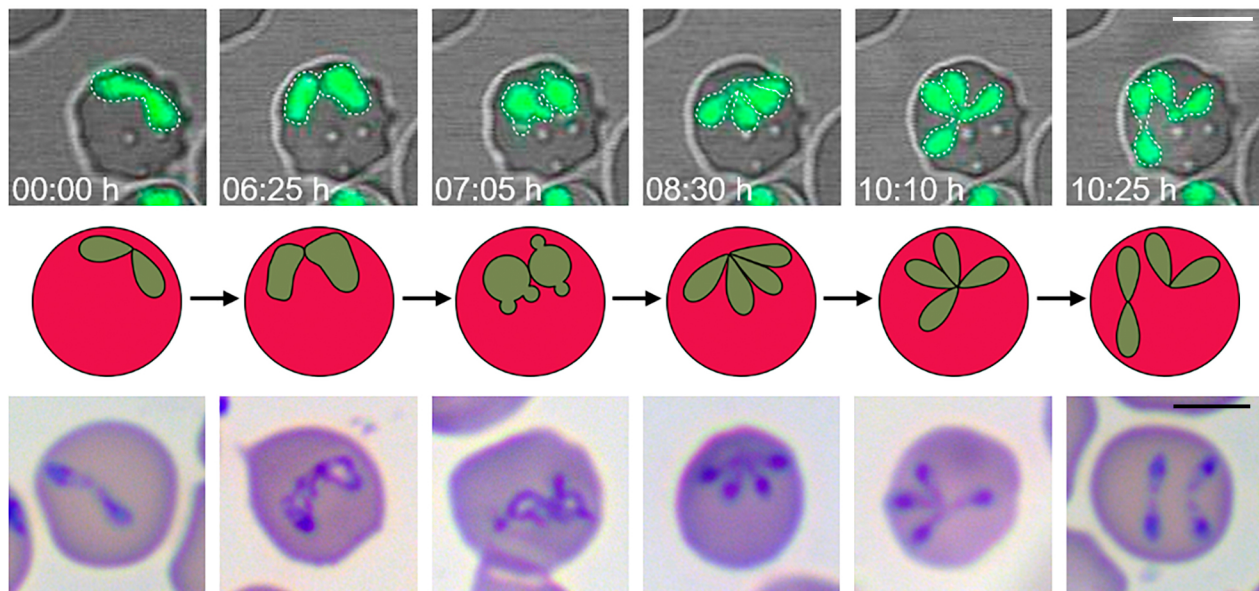

Supplement: FIG S8 [file mSphere.00928-20-sf008.pdf]

**a**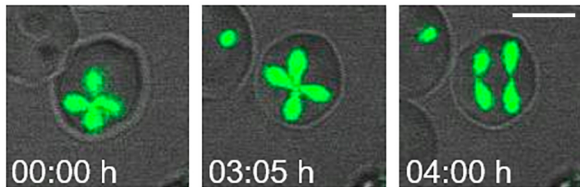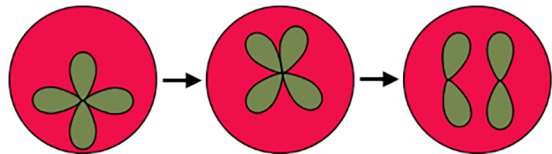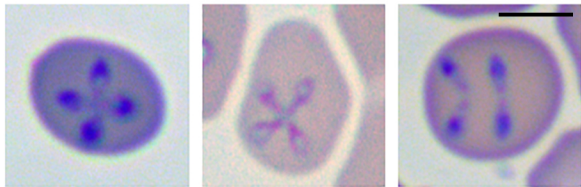**b**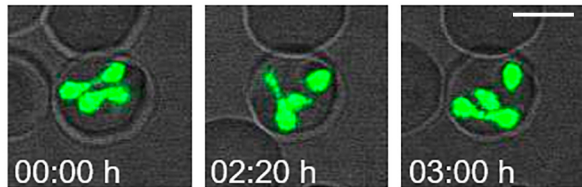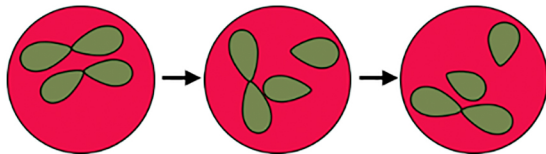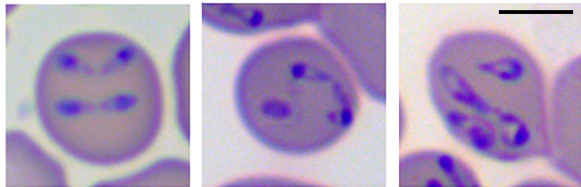

Supplement: FIG S9 [file mSphere.00928-20-sf009.pdf]

**a**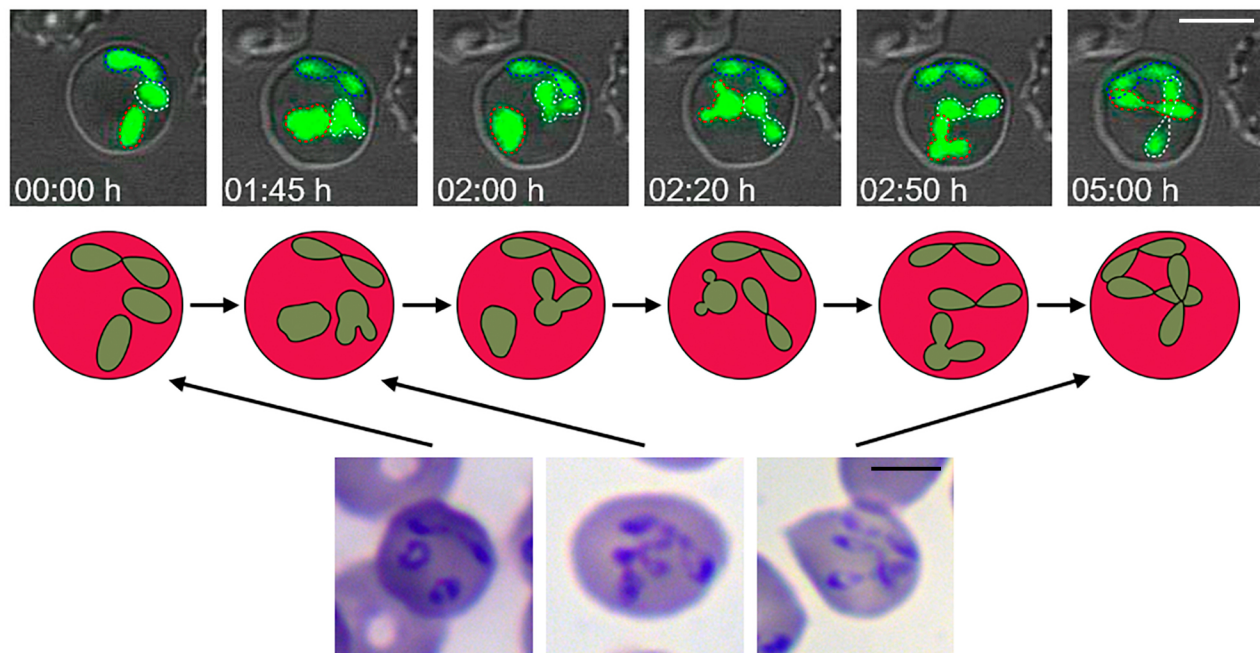**b**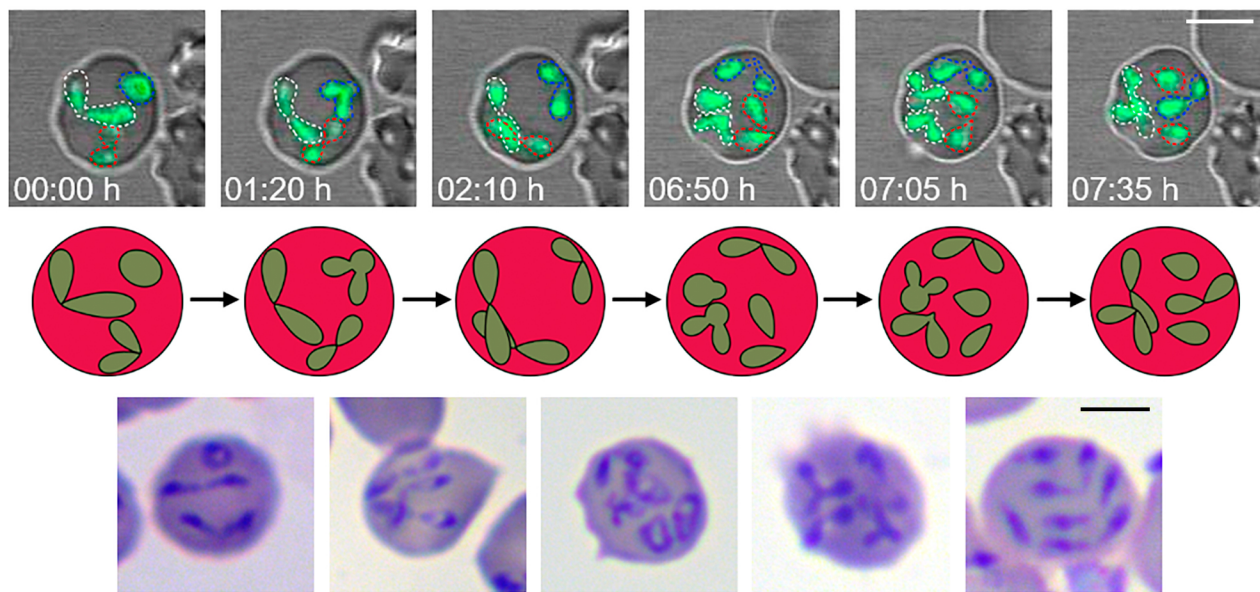

Supplement: FIG S10 [file mSphere.00928-20-sf010.pdf]
